# Supplementary material for: Full electrical manipulation of perpendicular exchange bias in ultrathin antiferromagnetic film with epitaxial strain
Source: Nat Commun. 2024 Jun 3;15:4734. doi: 10.1038/s41467-024-49214-z (PMC11148026; doi:10.1038/s41467-024-49214-z)
Supplement: Supplementary file 1 — Supplementary Information [file 41467_2024_49214_MOESM1_ESM.pdf]

Supporting Information to

Full electrical manipulation of perpendicular exchange bias in ultrathin antiferromagnetic film with epitaxial strain

Jie Qi, Yunchi Zhao, Yi Zhang, Guang Yang, He Huang, Haochang Lyu, Bokai Shao, Jingyan Zhang, Jialiang Li, Tao Zhu, Guoqiang Yu, Hongxiang Wei, Shiming Zhou, Baogen Shen, and Shouguo Wang

-contents-

Supplementary Note 1: Robustness and reproducibility of the PEB

Supplementary Note 2: IrMn-thickness dependence of the PEB fields

Supplementary Note 3: Epitaxial strain-dependent PEB

Supplementary Note 4: Atomistic simulation

Supplementary Note 5: The role of SOT in the PEBS process

Supplementary Note 6: Current density- and temperature-dependence of PEBS

Supplementary Note 7: Field-free SOT switching in Pt/Cr/Co/IrMn

Supplementary Note 8: Double-biased PEBS process by pulsed current

Supplementary Note 1. Robustness and reproducibility of the PEB

The experimental results of the AHE for five batches of the samples with the same structure of Pt (3 nm)/Co (1 nm)/$IrMn_3$ (2 nm) were summed up. The corresponding perpendicular exchange bias field is extracted from ten devices located at different positions of each sample to confirm the robustness and reproducibility of the considerable perpendicular exchange bias effect as shown in Fig. S1.

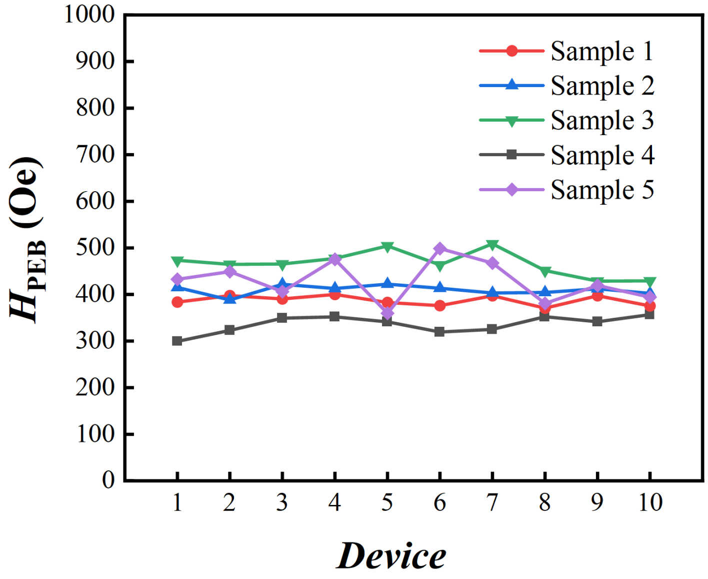

Figure S1. | Perpendicular exchange bias effect of five samples. H_{PEB} of five different samples extracted from ten devices located at different positions of each sample.

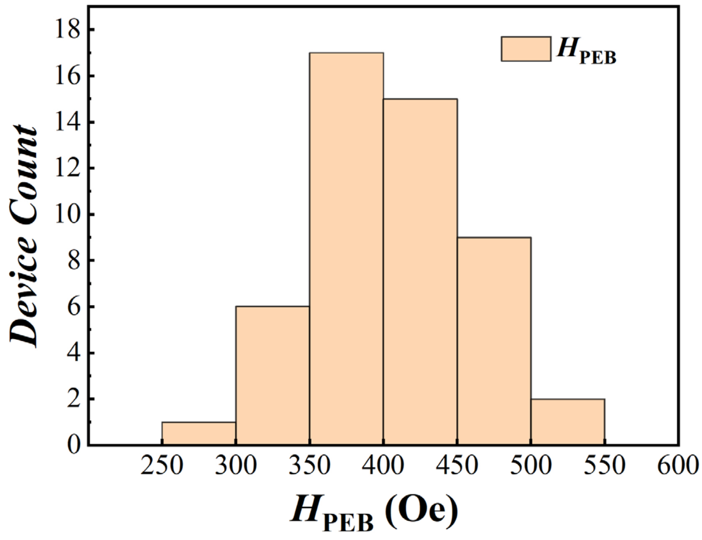

Figure S2. | Statistical H_{PEB} of the five samples. Statistical analysis of the perpendicular exchange bias fields for various devices of the five samples.

The distribution of perpendicular exchange bias fields for the fifty devices in the different samples is listed below. Statistical analysis of the perpendicular exchange bias fields for various devices was performed, as shown in Fig. S2. The majority of devices exhibited exchange bias field distributions centered around 400 Oe, suggesting that the considerable perpendicular exchange bias effect observed in the Pt (3 nm)/Co (1 nm)/$IrMn_3$ (2 nm) structure is reproducible and robust.

Supplementary Note 2. IrMn-thickness dependence of the PEB fields

The critical thickness of the antiferromagnetic IrMn layer to achieve a perpendicular exchange bias effect at room temperature was further identified. The dependence of H_{PEB} on t_{IrMn} is shown in Fig. S3, indicating that the perpendicular exchange bias effect emerges with t_{IrMn} larger than 1.5 nm. Consequently, the critical thickness of the antiferromagnetic IrMn layer to achieve a perpendicular exchange bias effect at room temperature is identified to be approximately 1.5 nm.

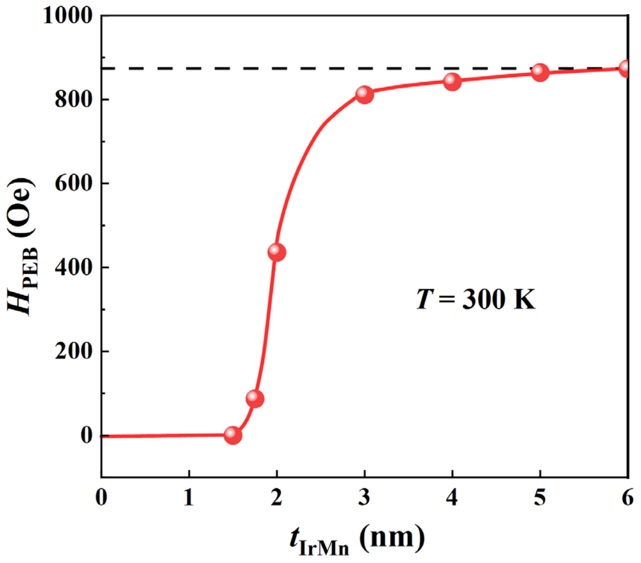

Figure S3. | t_{IrMn} dependence of the PEB fields. The curves of H_{PEB} vs t_{IrMn} at room temperature. The black dashed line marks the saturated H_{PEB} for the Pt/Co/$IrMn_3$ multilayers.

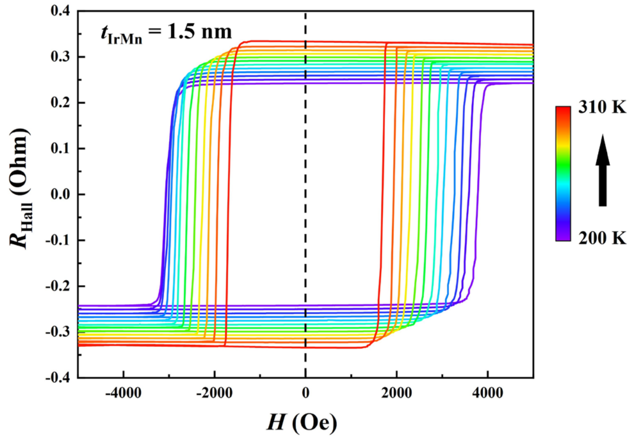

Figure S4. | Anomalous Hall effect of the sample with t_{IrMn} = 1.5 nm. AHE loops for the Pt/Co/$IrMn_3$ multilayer with t_{IrMn} = 1.5 nm at different temperatures ranging from 200 K to 310 K, respectively.

The result can also be verified by the temperature dependence of H_{PEB} of the Pt/Co/IrMn (1.5 nm) multilayer. The field-cooling process to 200 K was performed on the sample with t_{IrMn} = 1.5 nm under a -5000 Oe magnetic field. Then the AHE loops of the sample with increasing temperatures ranging from 200 K to 310 K were measured as shown in Fig. S4. The extracted H_{PEB} as a function of T was exhibited in Fig. S5. The H_{PEB} decreased with the increasing temperature and was negligible at 300 K, demonstrating that the transition thickness of the antiferromagnetic IrMn layer at room temperature is approximately 1.5 nm.

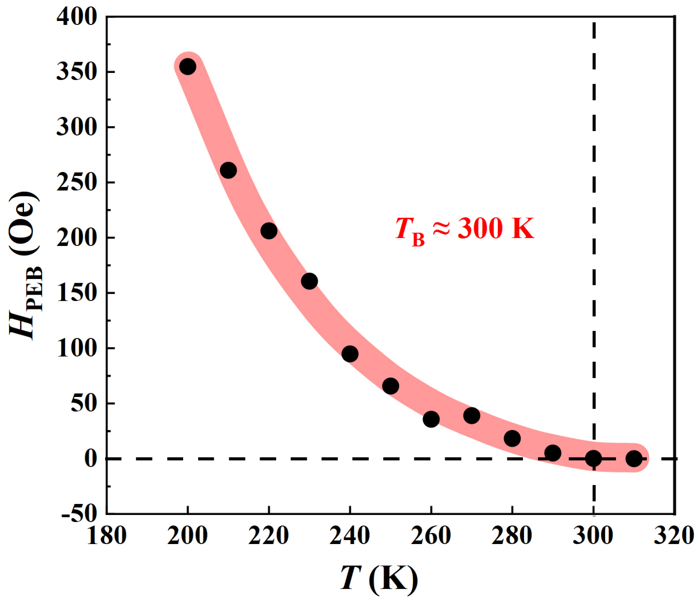

Figure S5. | Temperature dependence of H_{PEB}. The curve of H_{PEB} as a function of T for the sample with t_{IrMn} = 1.5 nm. The black dashed lines mark the blocking temperature for the sample.

Supplementary Note 3. Epitaxial strain-dependent PEB

a

b

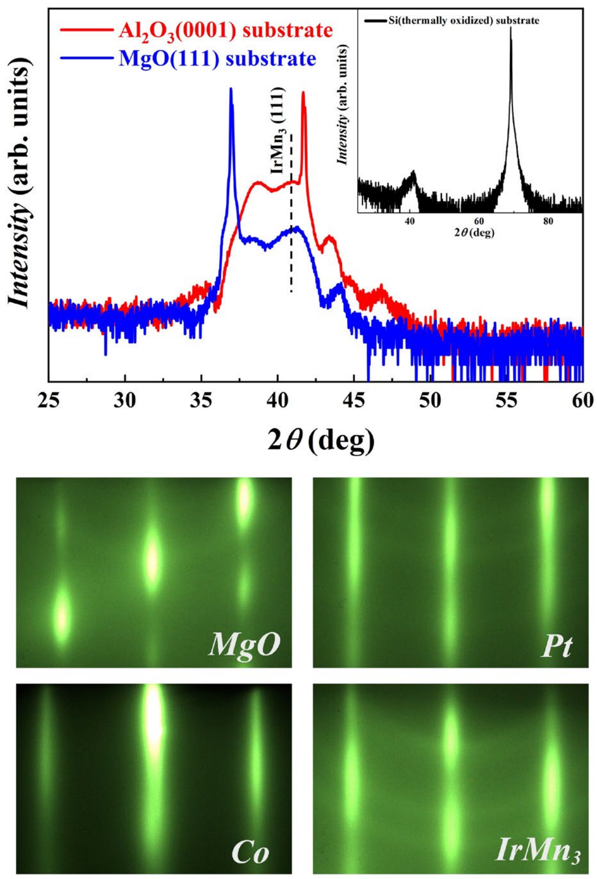

Figure S6. | Structural characterization of the control samples. a, XRD θ-2θ scans for the samples with Al_2O_3 (0001) (the red line) and MgO (111) (the blue line) substrates. All peaks were calibrated based on a standard powder diffraction file card of substrates for XRD with Cu-K_α radiation. The black dashed line marks the 2θ of the $IrMn_3$ (111) peak. Inset: XRD θ-2θ scan for the sample with thermally oxidized Si substrate. **b**, RHEED patterns for MgO substrate, Pt, Co, and $IrMn_3$ layers with incident electron beam parallel to the $[1\bar{1}0]$ direction of MgO substrate.

Two control samples consisting of the Pt (3)/Co (1)/$IrMn_3$ (2) (in nm) structure deposited on MgO (111) and Si substrates (thermally oxidized) were prepared to clarify the correlation between the epitaxial strain and the perpendicular exchange bias effect. Fig. S6(a) illustrates the XRD patterns of the samples with the same configuration, Pt/Co/$IrMn_3$, deposited on Al_2O_3 (0001) (the red line) and MgO (111) (the blue line) substrates on a logarithmic scale, respectively. A similar single-crystalline structure of $IrMn_3$ can be found in both samples with different lattice mismatches. Accordingly, the

high intensity of the diffraction peak of $IrMn_3$ for the sample with Al_2O_3 (0001) sample corresponds to an excellent single-crystalline structure with few defects. In contrast, the sample deposited on thermally oxidized Si substrate exhibits only a weak diffraction peak attributed to multiple contributions from textured Pt and $IrMn_3$, as shown in the inset, accompanied by a large amount of grain boundaries owning to the low degree of texture in a polycrystalline structure.

The RHEED patterns of the films deposited on the MgO (111) substrate were shown in Fig. S6(b) with sharp and continuous diffraction streaks, demonstrating the formation of the epitaxial structure of the Pt/Co/$IrMn_3$ multilayers on the MgO substrate.

Figure S7 shows the rocking curves (ω-scans) of $IrMn_3$ (111) for the Pt/Co/$IrMn_3$ structures deposited on various substrates, whereby all the peaks are symmetrical and centered at the Bragg position of $IrMn_3$ (111). Corresponding full width at half maximum (FWHM) are 0.34°, 1.49°, and 14.93° for samples with Al_2O_3 (0001) (the red line), MgO (111) (the blue line), and thermally oxidized Si (the black line) substrates, respectively. Compared to the lattice mismatch of Pt (111) on the Al_2O_3 (0001) substrate (0.95%), that of Pt on the MgO (111) substrate escalates to -6.84% resulting in the introduced defects and a reduction in the epitaxial quality of the entire epitaxial structure on MgO (111) substrate. For the polycrystalline Pt/Co/$IrMn_3$ deposited on the thermally oxidized Si substrate, the rocking curve exhibits wide distribution due to the low degree of texture in the polycrystalline structure possessing massive grain boundaries. Considering the relaxation of epitaxial strain surrounding the defects or grain boundaries (refer to ref. 56 and 57 in the manuscript), the epitaxial strain in $IrMn_3$ can be evaluated by the FWHM. As a result, the $IrMn_3$ layers deposited on three substrates can be considered in distinct strain states. Specifically, the epitaxial strain in $IrMn_3$ layers on Al_2O_3 (0001), MgO (111), and thermally oxidized Si substrates is degressive.

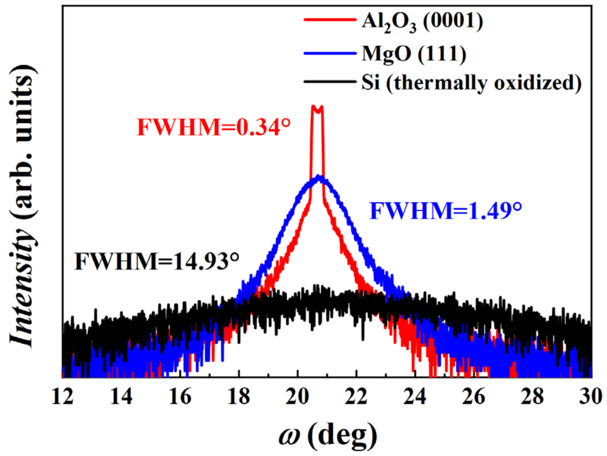

Figure S7. | Epitaxial strain in the ultrathin $IrMn_3$ layers. ω-scans measured at the Bragg position of $IrMn_3$ (111) for samples with Al_2O_3 (0001), MgO (111), and thermally oxidized Si substrates.

The anomalous Hall measurements were performed to evaluate the perpendicular exchange bias field of the control samples, as portrayed in Fig. S8. The epitaxial Pt/Co/$IrMn_3$ deposited on the MgO substrate exhibits an H_{PEB} of 67 Oe, whereas the H_{PEB} of the polycrystalline sample is negligible. Consequently, the degressive epitaxial strain in the $IrMn_3$ layers with Al_2O_3 (0001), MgO (111), and thermally oxidized Si substrates contributes to the decreasing H_{PEB} for the three samples, corresponding to the simulation results, which indicates a clear influence of the epitaxial strain on the perpendicular exchange bias effect for the ultrathin $IrMn_3$ layer.

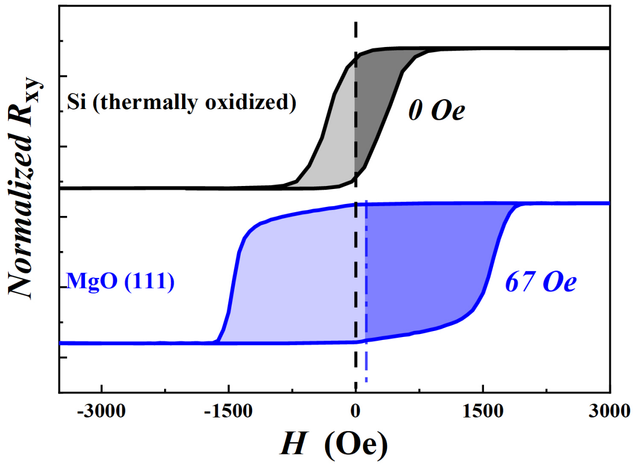

Figure S8. | Exchange bias effect of the control samples. Normalized AHE loops for the samples deposited on MgO (111) (the blue curve) and thermally oxidized Si substrates (the black curve).

Supplementary Note 4. Atomistic simulation

Our simulations were performed using an atomistic spin model with the VAMPIRE software package, where the energy of the system is described by a classical Heisenberg Hamiltonian ($\mathcal{H}$):

$$\mathcal{H} = -\sum_{i<j} J_{ij}\mathbf{S}_{\mathrm{i}} \cdot \mathbf{S}_{\mathrm{j}} - \frac{k_N}{2}\sum_{i\neq j}^{z}\left(\mathbf{S}_{\mathrm{i}} \cdot \mathbf{e}_{\mathrm{ij}}\right)^2 - \sum_i k_u(\mathbf{S}_{\mathrm{i}} \cdot \mathbf{e}_{\mathrm{z}})^2 - \sum_i \mu_S \mathbf{S}_{\mathrm{i}} \cdot \mathbf{B} \quad (1)$$

The first term above corresponds to the exchange energy, where $\mathbf{S}_i$ and $\mathbf{S}_j$ are normalized spin vectors on site i and j, respectively. J_{ij} is the effective exchange interactions between spin i and j, which were limited to nearest and next-nearest neighbors for the AFM layer. The second term represents the Néel anisotropy energy of the AFM layer, in which k_N describes the Néel anisotropy constant (-4.2×10^{-22} J/link) (refer to ref. 38 in the manuscript), and $\mathbf{e}_{ij}$ is the unit vector from site i to site j. The third term is the uniaxial anisotropy energy of the FM layer or the E_{MCA} of the AFM layer, where k_u is the uniaxial anisotropy constant (6.0×10^{-24} J/atom) for the Co layer. The last term describes the Zeeman energy, where μ_s is the atomic spin moment of Mn (2.65 μ_B) (refer to ref. 38 in the manuscript) or Co (1.72 μ_B) (refer to ref. 58 in the manuscript) atoms.

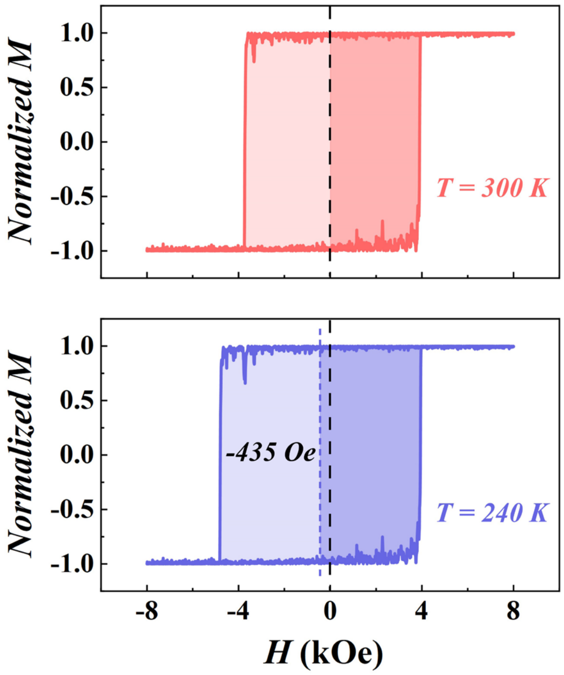

Figure S9. | Blocking temperature of the 2-nm-thick $IrMn_3$ layer without epitaxial strain. Simulated hysteresis loops with E_{MCA} = 0 J/atom at 300 K and 240 K of the Co/$IrMn_3$ bilayers.

Figure S9 presents the simulated hysteresis loops with E_{MCA} = 0 J/atom at 300 K

and 240 K. The H_{PEB} is extracted to be 0 Oe at 300 K, corroborating the conclusion that the anisotropy energy in the ultrathin $IrMn_3$ layer cannot overcome the interfacial exchange energy at the interface, manifesting as the absence of H_{PEB}. Additionally, the H_{PEB} emerges at 240 K with a value of -435 Oe, indicating that the blocking temperature of the system with E_{MCA} = 0 J/atom is approximately 240 K.

Supplementary Note 5. The role of SOT in the PEBS process

Additionally, the role of current-induced SOT in the PEBS process was investigated. In the sample with Pt/Co/IrMn structure, the magnetization direction of Co was fixed in the "up" state utilizing a magnetic field, and then a positive and a negative pulsed current was injected to change the polarization direction of the spin current, respectively. It was indicated by the AHE curves of the sample that the perpendicular exchange bias effect remains unchanged and consistently exhibits a negative state, irrespective of the direction of the pulsed current, as shown in Fig. S10a and b. Similarly, when the magnetization of Co was fixed in the "down" state, the perpendicular exchange bias remained positive state, as shown in Fig. S10c and d. The results substantiate that in the epitaxial Pt/Co/IrMn structure, current-induced SOT does not play a predominant role in the PEBS process.

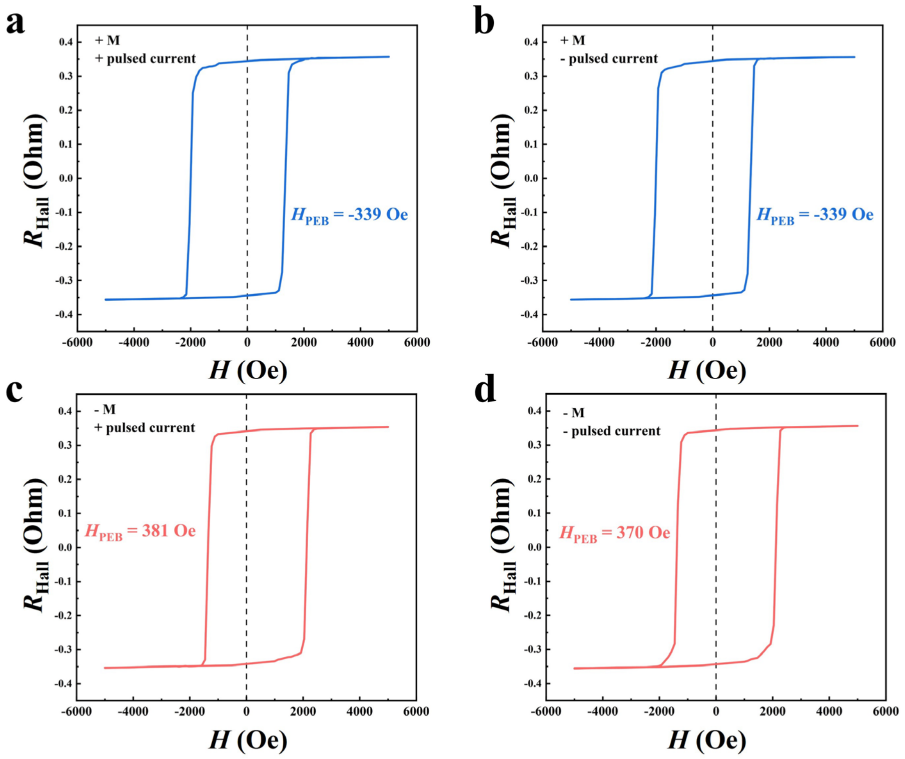

Figure S10. | Current-direction dependence of the PEBS. AHE loops for the Pt/Co/$IrMn_3$ multilayer after opposite pulsed currents with the magnetization of the Co layer set as "up" (a, b) and "down" (c, d) states, respectively.

Supplementary Note 6. Current density- and temperature-dependence of PEBS

The current-induced switching behavior of PEB in Pt/Co/$IrMn_3$ multilayers with varying pulsed current conditions was investigated to offer a comprehensive understanding of the underlying mechanism. Specifically, upon applying a pulsed current accompanied by a positive (negative) field along the out-of-plane direction, the exchange bias was reset to the negative (positive) state. Then the $\mathbf{m}_{Co}$ was set to the 'down' ('up') state, and subsequently, the increasing pulsed current with a duration of 80 μs was injected along the current channel without external fields. Fig. S11 exemplified the AHE loops of the sample after different magnitudes of the pulsed current with the $\mathbf{m}_{Co}$ set as "down" (Fig. S11a) and "up" (Fig. S11b) states, respectively, indicating the progressive switching behavior of the exchange bias between negative and positive states.

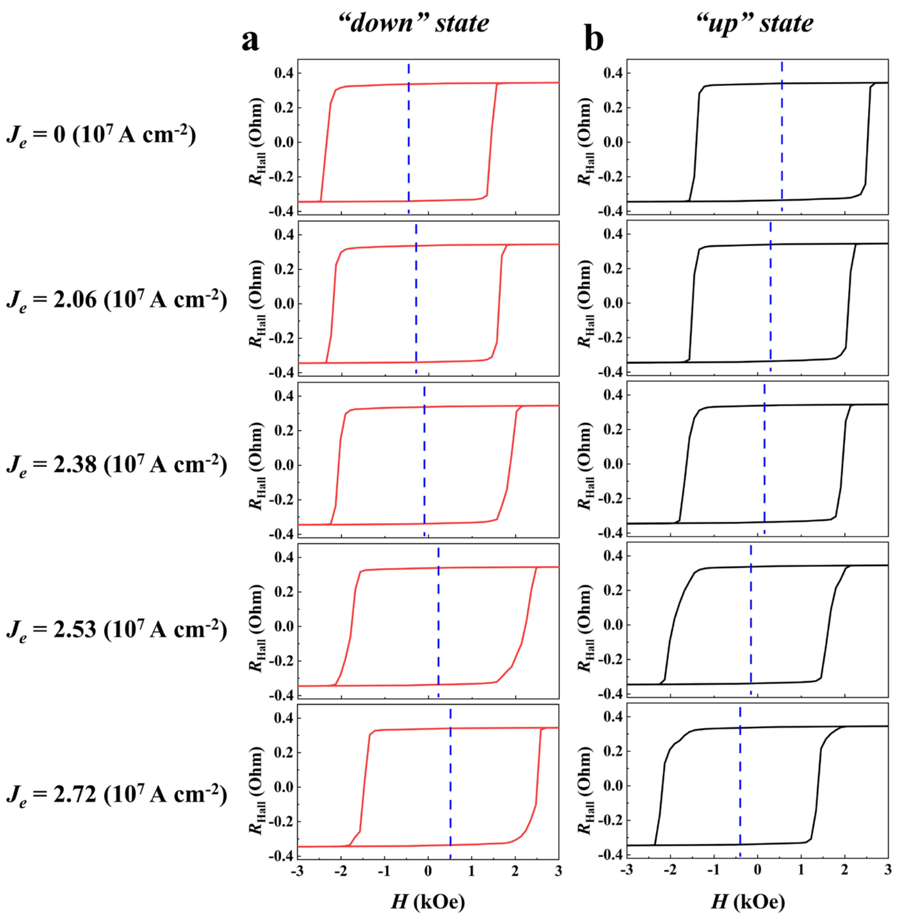

Figure S11. | Current density-dependence of PEBS. AHE loops for the Pt/Co/$IrMn_3$ multilayer after different pulsed currents with the magnetization of the Co layer set as "down" (**a**) and "up" (**b**) states, respectively. The blue dashed lines mark the H_{PEB} of each curve.

The relationship between the perpendicular exchange bias effect and the pulsed current is shown in Fig. S12. It indicates that the H_{PEB} changes gradually by increasing pulsed current in the absence of the external magnetic fields. With increasing pulsed current density, the exchange bias field changes from the negative direction to the positive direction, fully reversing its sign at a current density of 2.7×10^7 A cm^{-2}.

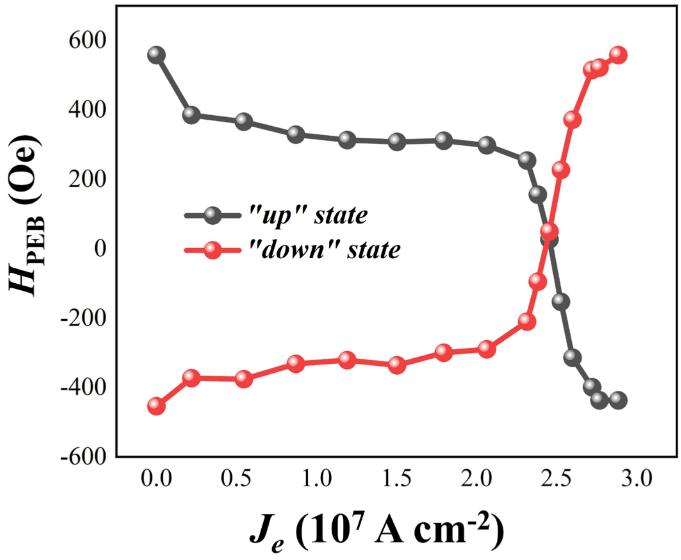

Figure S12. | Current density-dependence of PEBS. The curves of H_{PEB} as a function of J_e with the $\mathbf{m}_{Co}$ reset as “up” (the black curve) and “down” (the red curve) states, respectively.

Additionally, the temperature rise during the current injection was measured as shown in Fig. S13. The red solid curve depicts the variation of resistivity in response to the application of pulsed currents. The temperature rise is calibrated using the relationship between resistivity and temperature (the blue data points). For a pulsed current of approximately 2.7×10^7 A cm^{-2} to achieve a complete PEBS process, the resistivity of the device increased from 59.62 μΩ cm to 66.37 μΩ cm, corresponding to a nonnegligible temperature increase from 294 K to 395 K during the pulsed current, as marked with the black dashed lines. Additionally, the hollow red data points, recorded after the cessation of the pulsed current, exhibit negligible change, suggesting that the resistivity of the sample can be restored after each pulsed current within 3 seconds.

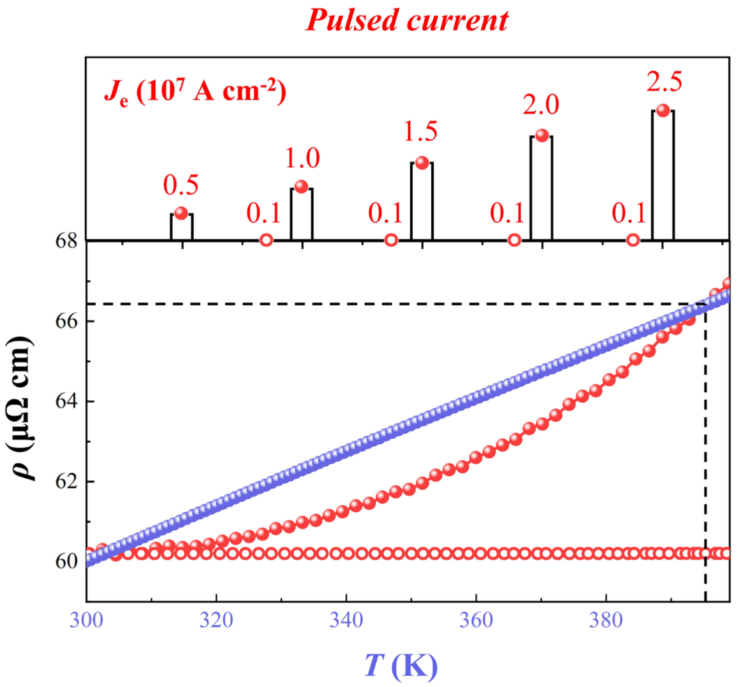

Figure S13. | Temperature rising originating from the pulsed current. The resistivity as a function of temperature (the blue solid points) and current density (the red solid points) curves for the Pt (3 nm)/Co (1 nm)/$IrMn_3$ (2 nm) structure. The red hollow data points represent the resistivity recorded after the cessation of each pulsed current. The black dashed lines mark the resistivity of the Pt (3 nm)/Co (1 nm)/$IrMn_3$ (2 nm) structure with a current density of 2.7×10^7 A cm^{-2} and the corresponding temperature of the device.

The blocking temperature (T_B) corresponds to the temperature above which the exchange bias effect vanishes. With the exchange bias effect of the sample reset as the positive saturated state, the AHE measurements were performed at different temperatures. Fig. S14 exemplified the Hall loops at 300 K (a), 330 K (b), 360 K (c), 370 K (d), 380 K (e), and 390 K (f), of which the perpendicular exchange bias field was derived as 489 Oe, 201 Oe, 24 Oe, 12 Oe, 0 Oe, and 0 Oe, respectively. The H_{PEB} decreased with the increasing temperature, and was negligible at 380 K as shown with the black curve in Fig. S16. Accordingly, the T_B of the Pt (3 nm)/Co (1 nm)/$IrMn_3$ (2 nm) structure was determined approximately to be 380 K.

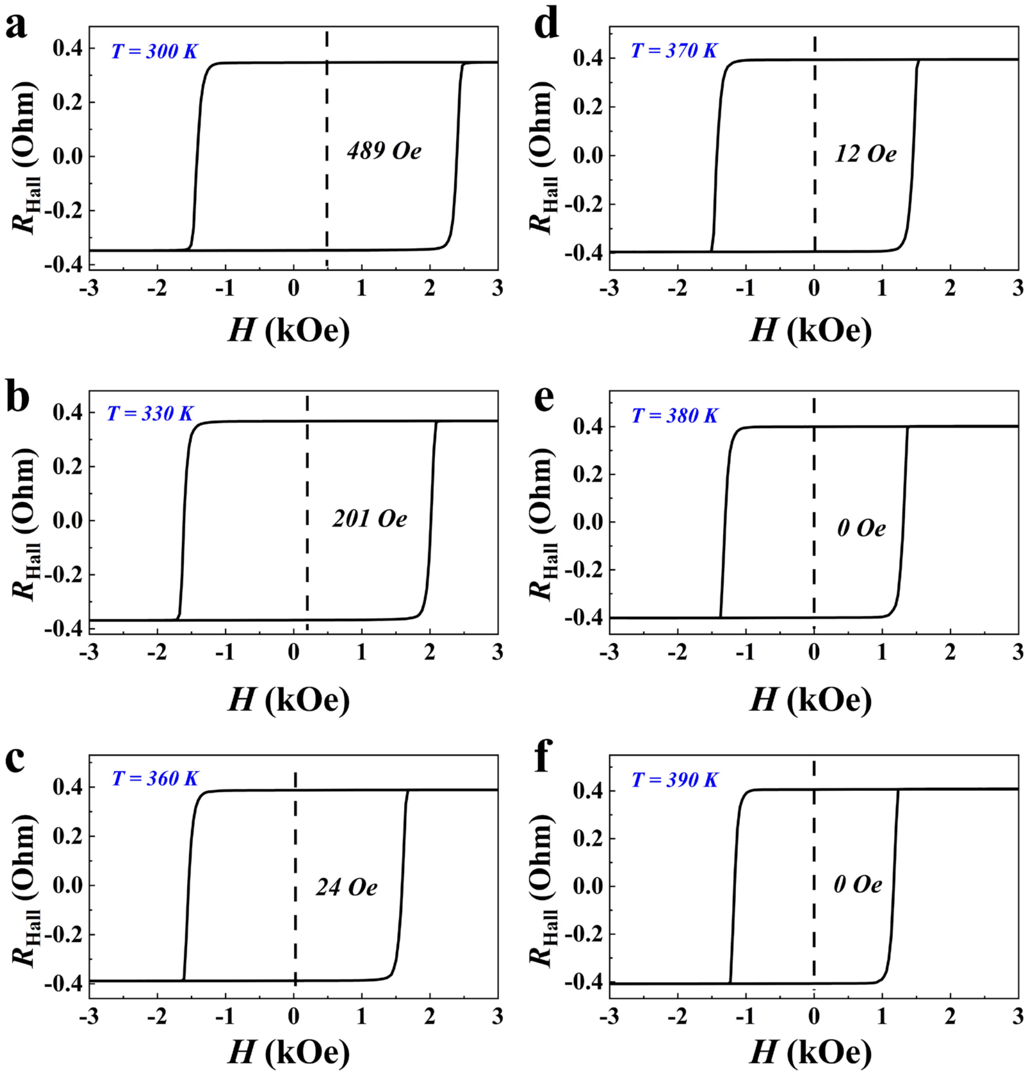

Figure S14. | Temperature-dependence of PEBS. a-f, AHE loops for the Pt/Co/$IrMn_3$ multilayer after different temperatures. The black dashed lines mark the H_{PEB} of each curve.

Interestingly, even without external magnetic fields during the pulsed current, the room-temperature exchange bias field is progressively switched, which exhibits a departure from both the PEBS driven by SOT and the conventional field-cooling process. In order to further clarify the physical intension of the PEBS process, the anomalous Hall curves obtained after cooling down to RT from different initial temperatures (zero-field cooling temperature, T_{ZFC}) without external magnetic fields were examined. The exchange bias effect of the Pt (3 nm)/Co (1 nm)/$IrMn_3$ (2 nm) structure was reinitialized as the positive saturated state, and the magnetization of the Co layer was reset to be "up" state. Afterwards, the sample was heated up to different

zero-field cooling temperatures (T_{ZFC}), and then was cooled down to 300 K in the absence of the external fields with the magnetization of Co maintaining the "up" state. The AHE loops of the sample going through different T_{ZFC} were acquired at 300 K. Fig. S15 exemplifies the AHE loops with the T_{ZFC} of 300 K (a), 320 K (b), 340 K (c), 360 K (d), 380 K (e), and 390 K (f), respectively. The corresponding H_{PEB} of the sample is derived as 489 Oe, 150 Oe, -225 Oe, -473 Oe, - 581 Oe, and -587 Oe, respectively. The H_{PEB} of the sample decreases with increasing the T_{ZFC} until the negative saturated state at about T_B (380 K), inconsistent with the conventional field-cooling performance, which requires an external field during the cooling process to determine the direction of the exchange bias effect.

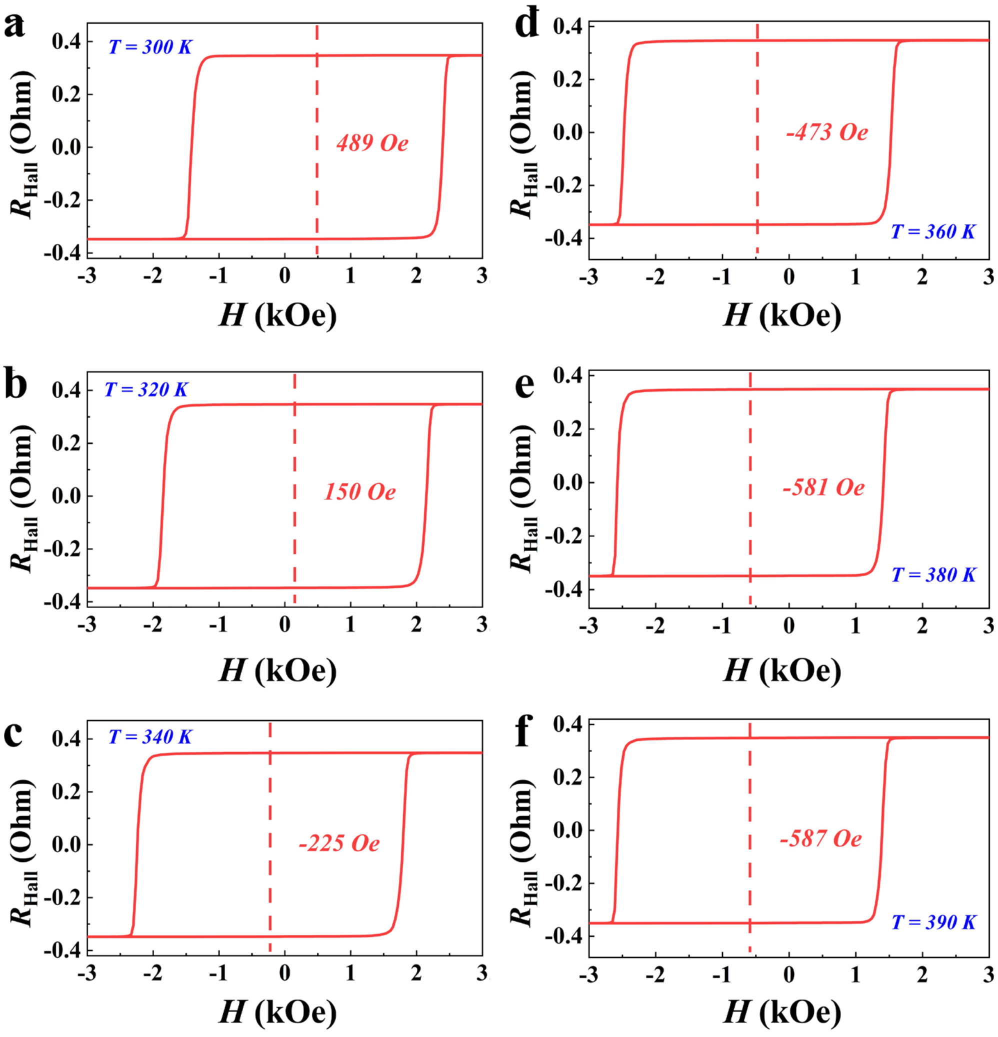

Figure S15. | T_{ZFC}-dependence of PEBS. a-f, AHE loops at room temperature for the Pt/Co/$IrMn_3$ multilayer with different T_{ZFC}. The red dashed lines mark the H_{PEB} of each curve.

The extracted exchange bias fields as a function of T_{ZFC} are shown in Fig. S16 as the red curve (with the system reset to the positive exchange bias state in advance, and the magnetic moment of Co was saturated to the "up" state prior to each curve measurement). Intriguingly, even in the absence of an external magnetic field during the cooling process, the corresponding room-temperature exchange bias field gradually switches toward negative saturation as the temperature increased, approximately reaching the negative saturation state near the T_B.

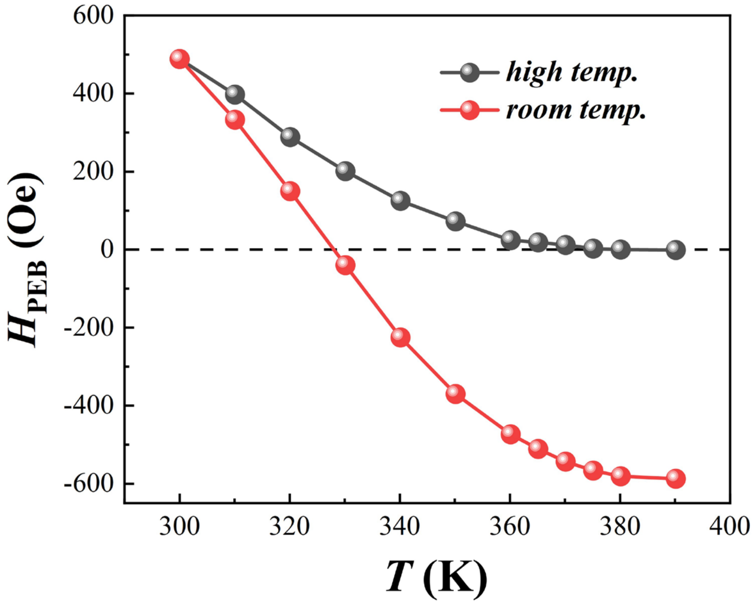

Figure S16. | Temperature- and T_{ZFC}-dependence of PEBS. The curve of H_{PEB} as a function of T (recorded at high temperature, the black curve) and T_{ZFC} (recorded at room temperature, the red curve).

Supplementary Note 7. Field-free SOT switching in Pt/Cr/Co/IrMn

In the context of the role of Cr wedge in the PEBS process, two potential mechanisms are considered. The introduction of a wedged structure may cause lateral inversion symmetry breaking, achieving field-free magnetization switching as reported in previous work (refer to ref. 63 in the manuscript). The other configuration is to introduce an in-plane component of the exchange bias field (refer to ref. 64 in the manuscript), which can serve as an alternative to the external magnetic fields, facilitating field-free magnetization switching.

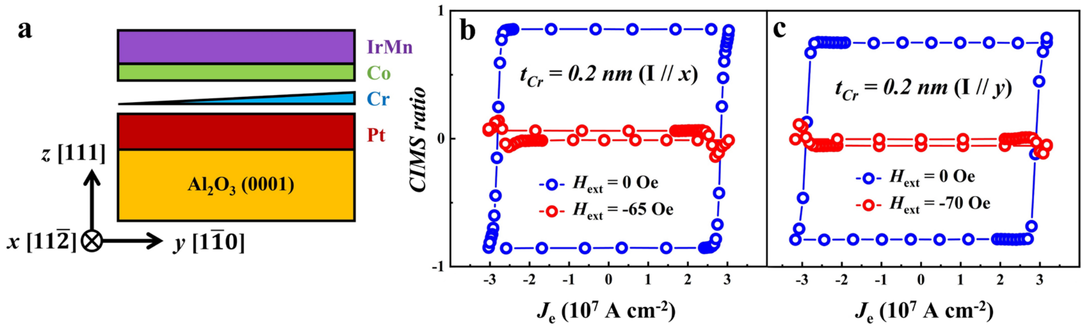

Figure S17. | Field-free magnetization switching. a, Schematic structure of the epitaxial Pt/Cr/Co/$IrMn_3$ multilayer. The current-induced magnetization switching at zero field (blue symbols) and non-zero field (red symbols) with the pulsed current injected perpendicular to (**b**) and along (**c**) the wedge direction, respectively.

Furthermore, experimental validation of these hypotheses was conducted. As illustrated in Fig. S17a, the *x*, *y*, and *z* axes are defined along [11$\bar{2}$], [1$\bar{1}$0], and [111] direction of the Pt layer, respectively. For the device with 0.2-nm-thick Cr insertion, the current-induced field-free magnetization switching could be realized with the current injected perpendicular to the wedge direction (*I* // *x*). Subsequent tests were performed to evaluate the current-induced magnetization switching behavior with the pulsed current injected along the wedge direction (*I* // *y*). The field-free magnetization switching behavior can also be observed as shown in Fig. S17c. The results suggest that the field-free switching in this structure is not attributed to the wedged structure. In addition, the effective fields for both configurations were identified by applying a negative in-plane magnetic field, under which the SOT-induced magnetization switching ratio is negligible. As shown in red symbols in Fig. S17b and c, the

corresponding effective fields were clarified to be 65 Oe and 70 Oe, respectively.

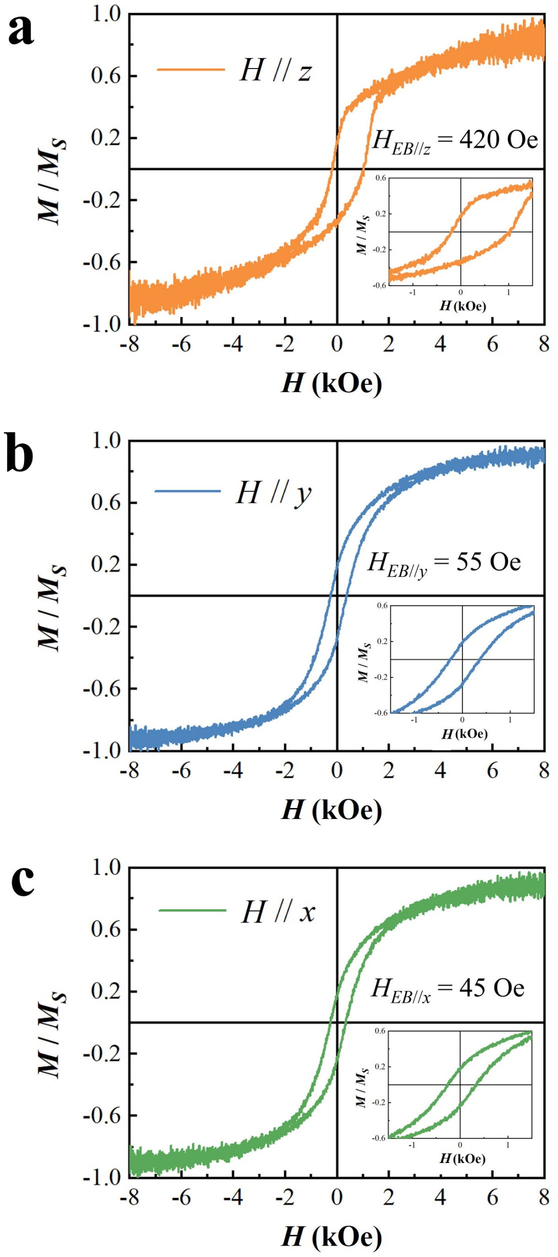

Figure S18. | In-plane and out-of-plane hysteresis loops. *M*-*H* loops for the sample with the structure of Pt (3 nm)/Cr (0.2 nm)/Co (1 nm)/IrMn (2 nm) with *H* along (**a**) *z* axis, (**b**) *y* axis, and (**c**) *x* axis, respectively.

To verify the origin of field-free magnetization switching behavior, a sample without the Cr wedge (sample structure: Pt (3 nm)/Cr (0.2 nm)/Co (1 nm)/IrMn (2 nm)) was deposited. The magnetic hysteresis loops of the sample were measured along both in-plane (*H* // *x*; *H* // *y*) and out-of-plane directions (*H* // *z*). With the magnetic field

perpendicular to the film, a perpendicular exchange bias field of 420 Oe was identified as shown in Fig. S18a, consistent with the AHE result observed in device D as shown in the manuscript. As shown in Fig. S18b and c, exchange bias fields of about 50 Oe exist along both the in-plane directions, which is comparable to the effective fields in the SOT-induced magnetization switching process (Fig. S17b and c). Consequently, the in-plane exchange bias fields result in symmetry breaking in the SOT-driven magnetization switching processes, thereby facilitating full electrical manipulation of the perpendicular exchange bias.

Supplementary Note 8. Double-biased PEBS process by pulsed current

As mentioned in the manuscript, the full electrical manipulation of the exchange bias effect from the negative saturated state to the positive saturated state can be achieved utilizing the field-free magnetization switching of Co by SOT.

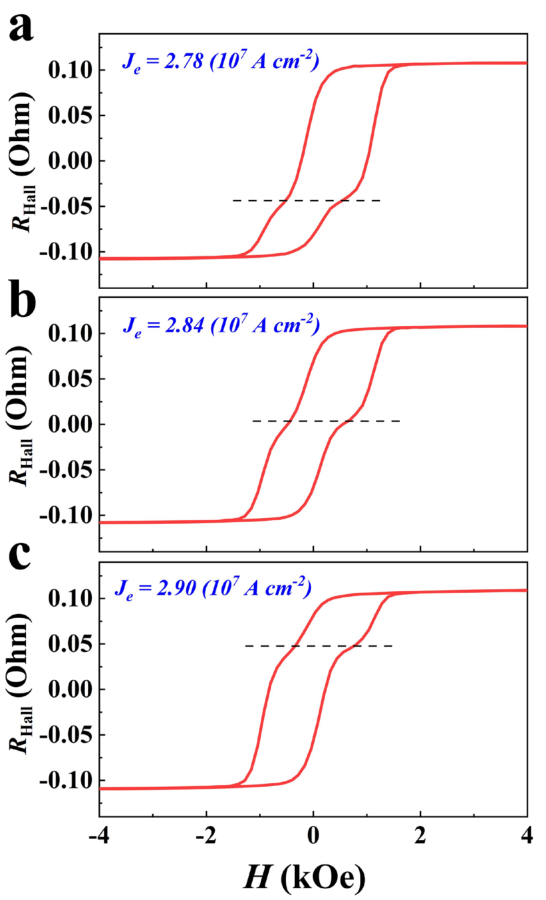

Figure S19. | Double-biased PEBS process. The double-biased AHE loops for the Pt/Cr (0.2 nm)/Co/$IrMn_3$ multilayer after the pulsed current density of 2.78×10^7 A cm^{-2} (**a**), 2.84×10^7 A cm^{-2} (**b**), and 2.90×10^7 A cm^{-2} (**c**), respectively. The black dashed lines mark the ratios of the negative and positive portions of the exchange bias effect.

Furthermore, a double-biased AHE loop can be induced by the pulsed current, as illustrated in Fig. S19. The exchange bias effect was reinitialized to be the positive saturated state. Thereafter, the pulsed currents with the current density of 2.78×10^7 A cm^{-2} (a), 2.84×10^7 A cm^{-2} (b), and 2.90×10^7 A cm^{-2} (c) were injected into the current channel, guaranteeing the switching ratios of Co were approximately 25%, 50%, and 75%, respectively, as illustrated in Fig. 4c of the manuscript. Concurrently, the AHE

loops of the sample were converted as the double-biased state, corresponding to the portions with the opposite exchange bias effect. Specifically, the ratio of the negative exchange biased portion with different pulsed current densities is approximately 25%, 50%, and 75%, respectively, in accordance with the switching ratios of Co. The results indicate that the ratio of the negative/positive portion in the double-biased loops can also be manipulated by the full electrical method, which arises from the incomplete switching behavior of the magnetization of the Co layer.
